# EpiDirect enables rapid bisulfite-free MGMT promoter methylation testing in IDH-wildtype glioblastoma with high agreement across established assays

**DOI:** 10.1093/noajnl/vdag255

**Published:** 2026-10-05

**Authors:** Attila Németh, Carmen Selignow, Annika May, Kai Schmid, Hildegard Dohmen, Marco Stein, Joshua Trautner, Daniel Haag, Felix Sahm, Till Acker

**Affiliations:** Institute of Neuropathology, Justus Liebig University Giessen, Giessen; Translational Neuroscience Network Giessen, Justus Liebig University Giessen, Giessen; Institute of Neuropathology, Justus Liebig University Giessen, Giessen; Institute of Neuropathology, Justus Liebig University Giessen, Giessen; Institute of Neuropathology, Justus Liebig University Giessen, Giessen; Institute of Neuropathology, Justus Liebig University Giessen, Giessen; Department of Neurosurgery, Justus Liebig University Giessen, Giessen; Department of Neuropathology, University Hospital Heidelberg, Heidelberg; Department of Neuropathology, University Hospital Heidelberg, Heidelberg; Department of Neuropathology, University Hospital Heidelberg, Heidelberg; Institute of Neuropathology, Justus Liebig University Giessen, Giessen; Translational Neuroscience Network Giessen, Justus Liebig University Giessen, Giessen

**Keywords:** EpiDirect, glioblastoma, MGMT promoter methylation

## Abstract

MGMT promoter methylation is a critical biomarker for predicting alkylating chemotherapy response in IDH-wildtype glioblastoma. However, the lack of an accepted reference detection method complicates clinical implementation. Current assays differ in their target CpG sites, analytical procedures, and classification thresholds. Here, we present an expanded paired analytical comparison of EpiDirect, a bisulfite-free quantitative PCR (qPCR) assay, with 4 established or emerging methods in clinical glioblastoma samples: pyrosequencing, MGMT-STP27 derived from EPIC methylation array data, methylation-specific PCR (MSP), and methylation-specific digital PCR (MS-dPCR).

Analysis of MGMT promoter methylation is included in current EANO recommendations for patients with IDH-wildtype glioblastoma.^1^ Despite its clinical relevance, no gold standard method has been generally accepted. Accurate and reproducible measurement remains challenging because MGMT assays differ in CpG coverage, analytical chemistry, and laboratory-specific classification thresholds and are further influenced by cellular heterogeneity, sequence variability, and FFPE sample quality. Commonly used methods include MSP, MS-dPCR, pyrosequencing, and MGMT-STP27 derived from Infinium MethylationEPIC array data.^2-4^ These methods require bisulfite conversion, adding a separate processing step that requires extra time and appropriate controls and may reduce DNA yield and quality. EpiDirect measures MGMT promoter methylation directly in untreated DNA. The method uses a primer containing modified nucleotides to enable selective binding to methylated DNA, followed by a duplex qPCR with an internal control.^5^ As with pyrosequencing reagents, EpiDirect reagents (PentaBase) are commercially available in a CE-IVD format. Here, we compared EpiDirect with MSP, MS-dPCR, MGMT-STP27, and pyrosequencing in clinical glioblastoma samples, thereby extending the original EpiDirect report, which compared EpiDirect with MSP and methylation-specific real-time quantitative PCR (MS-qPCR).

EpiDirect was locally calibrated for the QuantStudio 3 platform using control DNA with defined methylation levels and verified in independent runs. Limit of blank and limit of detection were determined, and a 5% decision threshold had been established before the present study using 10 precharacterized specimens. This threshold was further confirmed in an External Quality Assessment. We analyzed 35 formalin-fixed, paraffin-embedded IDH-wildtype glioblastoma samples by EpiDirect, MSP, MS-dPCR, pyrosequencing, and MGMT-STP27. EpiDirect measurements were performed in technical triplicate. Percentage methylation was calculated from the mean ΔCt after local calibration. Samples with an initial result between 4% and 10% methylation were reanalyzed in an independent run. Selected samples outside this interval were included as inter-run reproducibility controls, independently of the other MGMT assay results. If the first 2 valid runs yielded results on opposite sides of the 5% decision threshold, a third independent run was performed. Methylation was defined as >8% for pyrosequencing based on the mean methylation of 4 CpG sites, and >9% for MS-dPCR; MSP and MGMT-STP27 were interpreted according to their established assay-specific criteria. Agreement was assessed by overall percent agreement with exact 95% confidence intervals, Cohen’s κ, and the exact McNemar test.

Pairwise agreement between EpiDirect and the 4 established assays ranged from 88.6% to 94.3% with 95% confidence intervals shown in Figure 1 and corresponding Cohen’s κ values of 0.77-0.89. Exact McNemar tests did not indicate significant directional discordance in any pairwise comparison. All 5 assays gave the same classification in 30 of 35 tumors. Most discrepancies involved results close to assay-specific thresholds. This also applied to Case #11, which showed an isolated EpiDirect-positive result close to the decision threshold, with a mean methylation level of 5.67%. Case #24 showed a marked and reproducible discrepancy: EpiDirect, MGMT-STP27, and MSP classified the tumor as methylated, whereas pyrosequencing and MS-dPCR classified it as unmethylated. TET-assisted pyridine borane sequencing beta (TAPSβ) analysis^6^ revealed approximately 30% methylation at the PyroSeq/MS-dPCR target CpGs and a deletion overlapping the forward-primer binding site, consistent with assay-specific target dropout.

**Figure 1.**
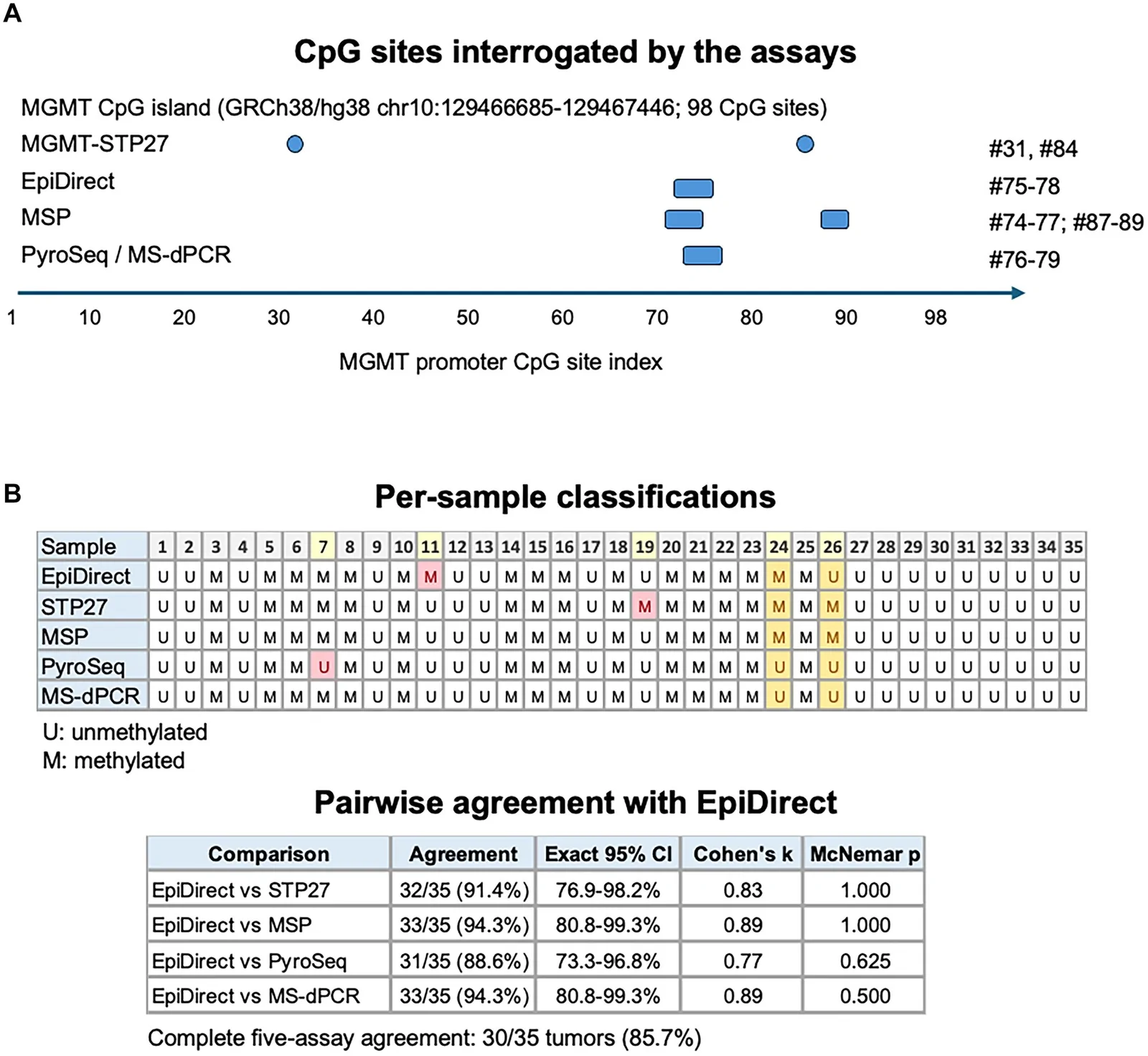
Comparison of MGMT promoter methylation results across 5 assays. (A) Schematic representation of MGMT promoter CpG sites interrogated by EpiDirect, MGMT-STP27, methylation-specific PCR (MSP), pyrosequencing, and methylation-specific digital PCR (MS-dPCR). (B) Per-sample methylation classifications with discordant results are highlighted, and the pairwise agreement between EpiDirect and the 4 established assays is shown. Discordant classifications in samples #7, #11, #19, and #26 involved values close to assay-specific decision thresholds; TET-assisted pyridine borane sequencing beta (TAPSβ) analysis indicated methylation at the PyroSeq/MS-dPCR target CpGs and a deletion overlapping the forward-primer binding site in sample #24. Underlying data are available at https://doi.org/10.22029/jlupub-20997.

EpiDirect generated a quantitative result directly from untreated DNA in approximately 2 h. MSP, MS-dPCR, pyrosequencing, and array-based MGMT-STP27 require bisulfite conversion followed by method-specific processing. Avoiding bisulfite conversion shortens the analytical workflow and reduces handling of limited tumor DNA. This is particularly relevant when a rapid standalone MGMT result is needed. Reanalysis is possible on the same or the following day. In our routine diagnostic experience, 3 of 38 glioblastoma cases had an initial methylation level of 5%-10% and required repeat testing. MGMT-STP27 remains useful when genome-wide methylation profiling is already performed for integrated tumor classification, but it cannot provide the same rapid standalone workflow. Costs for genome-wide methylation profiling were approximately €250-€500/case, versus €50-€100 for EpiDirect, pyrosequencing, and MS-dPCR and €10-€20 for MSP. EpiDirect combines a short, technically simple procedure with high agreement across established MGMT assays. The remaining discrepancies fit with the known assay dependence of MGMT classification, including differences in CpG coverage, decision thresholds, and primer-site alterations. The paired comparison also shows where current MGMT assays agree and where classification remains method-dependent in routine glioblastoma material. A particularly challenging issue is threshold definition to support decision-making algorithms, a topic discussed at length in recent publications.^2^^,^^3^ Dichotomization of a continuous biological signal inevitably reduces information and may contribute to assay-dependent classification, particularly for values close to the respective decision thresholds. For routine diagnostic laboratories, participating in External Quality Assessments is an important way to determine whether local threshold values yield concordant classification on defined test material, although clinical equivalence across methods ultimately requires validation in cohorts with outcome data.

Taken together, our findings are supportive of analytical non-inferiority of EpiDirect for MGMT promoter methylation testing in IDH-wildtype glioblastoma. Due to the small cohort size and the immaturity of survival data, prospective validation in larger cohorts with clinical outcome correlation is warranted.

## Data Availability

Data are available at https://doi.org/10.22029/jlupub-20997.
